# Prevalence and correlates of depression and anxiety among patients with tuberculosis at WolaitaSodo University Hospital and Sodo Health Center, WolaitaSodo, South Ethiopia, Cross sectional study

**DOI:** 10.1186/s12888-015-0598-3

**Published:** 2015-09-14

**Authors:** Bereket Duko, Abebaw Gebeyehu, Getnet Ayano

**Affiliations:** 1 Department of Psychiatry, College of Medicine and Health Sciences, Hawassa University, P.O.Box 1560, Hawassa, Ethiopia; 2 Institute of Public Health, College of Medicine and Health Sciences, University of Gondar, Gondar, Ethiopia; 3 Research and Training directorate, Amanuel Mental Specialized Hospital, Addis Ababa, Ethiopia

## Abstract

**Background:**

Anxiety and depression are frequently and highly occurring mental disorders in patients with tuberculosis. When depression and anxiety co-morbid with tuberculosis, it leads to poor adherence to anti TB medication, which is important barrier to global control of tuberculosis & increases the risk of morbidity and mortality due to TB. Cross sectional study was conducted to assess prevalence and correlates of depression and anxiety among patients with TB at WolaitaSodo University Hospital and Sodo Health Center, WolaitaSodo, Ethiopia.

**Methods:**

Institution based cross-sectional study was conducted in 2014.A total of 417 TB patients, who had regular follow up at WolaitaSodo University Hospital and Sodo Health Center, WolaitaSodo, South Ethiopia, were recruited to assess depression and anxiety and its associated correlates. Depression and anxiety were assessed through face to face interviews by trained psychiatry nurses using the hospital anxiety and depression scale (HADS). Correlates for depression and anxiety were assessed using a structured questionnaire, Oslo social support scale and TB stigma Scale.

**Results:**

The prevalence of depression and anxiety among patients with TB were 43.4 % (181) and 41.5 % (173) respectively. When we adjusted for the effect of potential confounding variables, patients who had co-morbid HIV infection [AOR = 5.90,(95 % CI: 2.34,15.93)], poor social support [AOR = 18.06, (95 % CI:11.21,25.45)] & perceived TB stigma [AOR = 10.86, (95 % CI:10.26,23.47)] were more likely to have depression as compared to individuals who had no co-morbid HIV infection, good social support and no perceived TB stigma respectively. Patients who had co-morbid HIV infection [AOR = 9.61,(95 % CI:3.56,25.96)], poor social support [AOR = 8.93,(95 % CI: 5.01,15.94)], perceived TB stigma [AOR = 3.11,(95 % CI:1.78,5.42)], being female [AOR = 1.72 (95 % CI: 1.06, 2.95)], current substance use[AOR = 4.88, (95 % CI: 1.79, 13.28)] and being on intensive phase of TB treatment [AOR = 1.91, (95 % CI: 1.08, 3.39)] were more likely to have anxiety as compared to individuals who had no co-morbid HIV infection, good social support, no perceived TB stigma, being male and being on continuous phase of TB treatment respectively.

**Conclusion:**

Developing guidelines and training of health workers in TB clinics is useful to screen and treat depression and anxiety among TB patients.

## Background

Tuberculosis is a chronic infectious disease caused by Mycobacterium tuberculosis. It is one of the leading causes of morbidity and mortality worldwide [1, 2]. According to WHO 2012 estimate, 2 billion people have latent TB, while another 3 million people worldwide die each year due to TB [3]. It remains a major global health problem & causes ill-health among millions of people each year and ranks as the second leading cause of death from an infectious disease worldwide, after HIV/AIDS [4]. According to WHO 2013 global TB control report, Ethiopia ranks 7th among the 22 high burden countries in the world and 2nd in Africa [5].

Depression is a common mental disorder that presents with depressed mood, loss of interest or pleasure, decreased energy, feelings of guilt or low self-worth, disturbed sleep or appetite, and poor concentration [6]. It is one of the leading causes of disease burden affecting 121 million people worldwide. Depression can lead to suicide. Suicide contributes for the loss of about 850,000 lives every year [7]. In general population, the life time risk of depression is 10 % to 25 % for women and 5 % to 12 % for men [8]. In Ethiopia, a study done in Addis Ababa showed that the life time prevalence is 2.7 % for depressive episodes, 0.2 % for recurrent episodes, 0.3 % for bipolar and 1.6 % for persistent mood disorder [9]. However, the prevalence of depression in those with chronic illness in the world is much higher, i.e. 25 % to 33 % [8].

Anxiety is a vague, subjective, non-specific feeling of uneasiness, apprehension, tension, (excessive nervousness) fears, and a sense of impending doom, irrational avoidance of objects or situation and anxiety attack [6]. The anxiety disorders make up one of the most common groups of psychiatric disorders and the National co-morbidity study reported that one of four persons met the diagnostic criteria for at least one anxiety disorder and that there is a 12-month prevalence rate of 17.7 percent [10].

Anxiety and depression are the most frequently occurring mental disorders in the general population [11]. Studies conducted in different countries on prevalence of depression and anxiety among TB patients shows that 46.3 % (anxiety), 47.2 % (depression) in Pakistan [11], 72.88 % (anxiety), 38.98 % (depression) in Romania [12], 40.67 % (anxiety), 9.93 % (depression) in Greece [13], 45 % (depression) in Nigeria [14], 61 % (depression in Kenya [15]. The studies indicate that there is high prevalence of depression and anxiety among TB patients compared to general population which is about 3–17 % [16], and 7 % to 82.3 % [17], respectively.

Moreover, depression often comes with symptoms of anxiety. These problems can become chronic or recurrent and lead to substantial impairments in an individual’s ability to take care of his or her everyday responsibilities [5]. The presence of depression and anxiety has a negative influence on quality of life, health care cost and self-care. This leads to decreased resistance to infections, so it adversely affects the patient’s compliance to TB treatment that can increase mortality from the disease [18]. Despite their known effect on the population, there is very little data available in the study area. Therefore, this study was planned to determine the prevalence and correlates of depression and anxiety among patients with TB at Wolaita Sodo University Hospital and Sodo health center, Wolaita Sodo, Ethiopia.

## Methods

### Study Setting and population

The study was a cross sectional design, conducted from April to May, 2014 in Wolaita Sodo University Hospital and Sodo Health Center, South Nation Nationalities regional state of Ethiopia. All adult patients (age ≥ 18) with tuberculosis who had regular follow were included in the sample. Critically ill patients were excluded from the study.

Among 948 TB patients who had regular follow up at TB clinics, 424 TB patients were recruited for the study. Study participants were included using systematic random sampling technique. Seven patients refused to participate in the study.

### Data collection

Data were collected by trained psychiatry nurses using pretested interviewer administered questionnaire. The data collection instrument had different components. The first part includes socio-demographic characteristics (age, education, occupation, marital status and others). Social support characteristics were collected by Oslo 3-item social support scale. Oslo 3-item social support scale is 3-item questionnaire commonly used to assess social support. It has the sum score scale ranging from 3 to 14 with three broad categories: “poor support” 3–8, “moderate support” 9–11 and “strong support” 12–14 [19]. It was reliable in the study (Cronbach's α = 0.91). Stigma felt by TB patients was collected by 12-item perceived TB stigma scale. It consisted of four-point Likert scale (strongly disagree, disagree, agree, strongly agree) questions concerning perceived isolation, shame, guilt and disclosure of the TB status. Item scores of the stigma questions were summed to construct a single stigma variable. Participants were classified as having or not having perceived stigma using the mean of the stigma variable as cut-off point [20]. The instrument was adopted and translated to Amharic language and back to English and highly reliable in the study (Cronbach's α = 0.89). An outcome variable (presence of anxiety and depression) was collected by Hospital Anxiety & Depression scale (HADS). HADS is a 14-item questionnaire, commonly used to screen for symptoms of anxiety and depression. The 14-item can be separated into two 7-item sub-scales for anxiety and depression. It was validated in Ethiopia and its internal consistency was 0.78 for anxiety, 0.76 for depression subscales and 0.87 for full scale. The scales use a cut off score for anxiety and depression of greater than or equal to 8 [21].

### Data Processing and Analyses

Data were analyzed using SPSS version 20. Bivariate analysis was done to see the association of each independent variable with the outcome variable. Potential confounders (important) variables were entered into binary logistic regression model to identify the effect of each independent variable with the outcome variables. A *p*-value of less than 0.05 was considered statistically significant, and adjusted odds ratio with 95 % CI was calculated to determine association.

### Ethical Consideration

Ethical clearance was obtained from the Research and Ethics Review Committee of the Institute of Public Health (University of Gondar) and Amanuel Mental Specialized Hospital. Permission letter was obtained from Wolaita zone Health Department and submitted to Sodo Health Center. Written informed consent was obtained from each study participant and they were informed about their rights to interrupt the interview at any time. Confidentiality was maintained at all levels of the study. Tuberculosis patients who were found to have moderate to severe depression and anxiety were referred to psychiatry clinics for further investigations.

## Results

### Socio-economic and demographic characteristics

A total of 417 participants were recruited for the study which makes the response rate 98.6 %. The mean ((± SD) age of the respondents was 34.52 (±11.01) years. Among the respondents, 291(69.8 %) were in age range of 25 – 49 years, 241(57.8 %) were male, 266 (63.8 %) were Wolaita ethnic background, 199 (47.7 %) were never married (single), 129 (30.9 %) were attended primary education, and 189 (45.3 %) were protestant religion followers. The median monthly income of the participants was 700 (Table 1).

**Table 1:** Distribution of TB patients at Wolaita Sodo University Hospital & Sodo Health Center, SNNPRS, Ethiopia, 2014

Variables		Frequency	Percent (%)
Age	18–24 years	88	21.1
	25–49 years	291	69.8
	> = 50 years	38	9.1
Sex	Male	241	57.8
	Female	176	42.2
Marital status	Married	182	43.6
	Unmarried	199	47.7
	Divorced	15	3.6
	Widowed	21	5.0
Education status	No formal education	78	18.7
	Primary education	129	30.9
	Secondary education	117	28.1
	Higher education	93	22.3
Religion	Protestant	189	45.3
	Orthodox	146	35.0
	Muslim	52	12.5
	Catholic	30	7.2
Ethnicity	Wolaita	266	63.8
	Amhara	62	14.9
	Gurage	69	16.5
	Others	20	4.8
Occupation status	Government employee	76	18.2
	Private employee	85	20.4
	Merchant	51	12.2
	Farmer	15	3.6
	House wives	52	12.5
	Jobless	49	11.8
	Students	89	21.3
Monthly income	<735 ETB	212	50.8
	735–1176 ETB	86	20.6
	> = 1176 ETB	119	28.6

### Clinical and psychosocial characteristics of the respondents

Two hundred eighty seven (68.8 %) patients were with diagnosis of pulmonary TB, 341 (81.8 %) were in new TB treatment category, 245 (59.2 %) had 6–12 months duration of illness, 270 (64.9 %) were in intensive phase of TB treatment, 229 (54.9 %) had good social support, 177 (42.4 %) had perceived TB stigma. From all study participants 49 (11.8 %) had co-morbid HIV illness, 30 (7.2 %) were currently substance (khat, cigarette and alcohol) users (Table 2).

**Table 2:** Description of clinical, psychosocial & substance use factors among patients with TB at Wolaita Sodo University Hospital and Sodo Health Center, SNNPRS, 2014.

Variables		Frequency	Percent (%)
Classification	Pulmonary TB	287	68.8
	Extra-pulmonary TB	130	31.2
Category of treatment	New	341	81.8
	Return after default	37	8.9
	Relapse/treatment after failure	39	9.4
Duration of illness	<6 months	25	6
	6–12 months	247	59.2
	> = 12 months	145	34.8
Phase of treatment	Intensive phase	270	64.7
	Continuation phase	147	35.3
Co-morbid chronic illness	HIV/AIDS	49	11.8
	Other chronic illness	24	5.8
	No co-morbid illness	344	82.5
Good social support	Yes	229	54.9
	No	188	45.1
Perceived TB stigma	Yes	177	42.4
	No	240	57.6
Substance (khat, cigarette & alcohol) use	Yes	30	7.2
	No	387	92.8

### Prevalence of depression and anxiety among TB patients

The prevalence of depression, anxiety and co-morbid depression and anxiety among TB patients were 43.4 %, 41.5 %, and 40.6 % respectively.

### Factors associated with depression & anxiety among patients with TB

Binary logistic regression analysis revealed that co-morbid chronic illness, good social support and perceived TB stigma were statistically significant with depression. Being female, phase of treatment, co-morbid chronic illness, good social support, perceived TB stigma, current substance (khat, cigarette & alcohol) use were statistically significant with anxiety (Table 3 & 4).

**Table 3:** Factors associated with depression among patients with TB at Wolaita Sodo University Hospital and Sodo Health Center, SNNPRS, Ethiopia, 2014

Explanatory Variables	Depression		COR,95 % (CI)	AOR,95 % (CI)
	Yes	No		
Age *n* = 417				
18–24 years	27	61	**1**	**1**
25–49 years	129	162	1.79, (1.08, 2.99)	0.78(0.30, 2.03)
> = 50 years	25	13	4.34, (1.94, 9.76)	1.09 (0.20, 5.92)
Educational status *n* = 417				
No formal education	48	30	3.20, (1.71, 5.99)	2.92 (0.91, 9.35)
Primary education	59	70	1.68, (0.97, 2.93)	2.58 ( 0.95 ,6.96)
Secondary education	43	74	1.16, (0.66, 2.06)	1.50 (0.57, 3.94)
Higher education	31	62	**1**	**1**
Classification *n* = 417				
Pulmonary TB	150	137	3.49, (2.19, 5.57)	1.68(0.97, 4.17)
Extra-pulmonary TB	31	99	**1**	**1**
Phase of treatment				
Intensive phase	127	143	1.53, (1.01, 2.31)	0.59 (0.29,1.23)
Continuous phase	54	93	**1**	**1**
Co-morbid chronic illness				
HIV/AIDS	34	15	3.83, (2.01, 7.29)	5.90(2.34, 15.93)**
Other chronic illness	19	5	6.41, (2.34, 17.59)	1.07 (0.37, 3.07)
No chronic illness	128	216	**1**	**1**
Good social support				
Yes	29	200	**1**	**1**
No	152	36	29.12, (17.10, 49.59)	18.06(11.21, 25.45)*
Perceived TB Stigma				
Yes	148	29	32.01, (18.62, 55.02)	10.86(10.26, 23.47)*
No	33	207	**1**	**1**

*significant association (*p*-value < 0.05) ** significant association (*p*-value < 0.01) Substance use = khat, cigarette and/or alcohol use .Other chronic illness = hypertension, renal diseases, cardiovascular diseases and diabetes

**Table 4:** Factors associated with anxiety among patients with TB at Wolaita Sodo University Hospital and Sodo Health Center, SNNPRS, Ethiopia, 2014

Explanatory Variables	Anxiety		COR,95 % (CI)	AOR,95 % (CI)
	Yes	No		
Sex *n* = 417				
Male	89	152	**1**	**1**
Female	84	92	1.56, (1.05, 2.31)	1.72, (1.06, 2.95)**
Educational status *n* = 417				
No formal education	45	33	3.01, (1.61, 5.64)	0.70,(0.23,2.11)
Primary education	54	75	1.59, (0.91, 2.79)	0.80,(0.32,1.99)
Secondary education	45	72	1.38, (0.78, 2.45)	1.31,(0.54,3.20)
Higher education	29	64	**1**	**1**
Job *n* = 417				
Government employed	28	48	**1**	**1**
Private employed	29	56	0.89, (0.47, 1.69)	0.79, (0.33, 1.93)
Merchant	20	31	1.11, (0.53, 2.29)	0.65, (0.23, 1.88)
Farmer	6	9	1.14, (0.37, 3.55)	0.70, (0.14, 3.56)
House wives	29	23	2.16, (1.05, 4.44)	1.24, (0.88, 6.23)
Jobless	26	23	1.94, (1.03, 4.02)	2.40, (0.62, 5.12)
Students	35	54	1.11, (0.59, 2.09)	2.34, (0.43, 3.32)
Category of treatment				
New	134	207	**1**	**1**
Return after default	16	21	1.18, (0.59, 2.34)	1.86,(0.71,4.87)
Relapse	23	16	2.22, (1.13, 4.36)	1.01,(0.39, 2.65)
Phase of treatment				
Intensive phase	130	140	2.25,(1.46, 3.45)	1.91,(1.081, 3.39)**
Continuous phase	43	104	**1**	**1**
Co-morbid chronic illness				
HIV/AIDS	42	7	11.95, (5.21, 27.43)	9.61, (3.56, 25.96)*
Other chronic illness	16	8	3.98, (1.66, 9.58)	2.62, (0.91, 7.58)
No chronic illness	115	229	**1**	**1**
Good social support				
Yes	36	193	**1**	**1**
No	137	51	14.40, (8.92, 23.26)	8.93, (5.01, 15.94)*
TB Stigma				
Yes	123	54	8.66, (5.54, 13.53)	3.12, (1.78, 5.42)**
No	50	190	**1**	**1**
Substance use				
Yes	21	9	3.607, (1.61, 8.09)	4.88, (1.79,13.28)*
No	152	235	**1**	**1**

* Significant association (I-value < 0.05) **- significant association (p-value < 0.01)

Substance use = khat, cigarette and/or alcohol use other chronic illness = hypertension, renal diseases, cardiovascular diseases and diabetes

## Discussion

### Prevalence and factors associated with depression among patients with Tuberculosis

This study revealed that the prevalence of depression was 43.4 %. The finding was similar with other studies carried out in Nigeria 41.9 % [22], in Ibadan Nigeria 45.5 % [14] and in Pakistan 46.3 % [11]. On the other hand, the current study finding was higher than the study done in Nigeria 27 % [23] and Greece 9.93 % [13] and lower than the study was done in Kenya 61 % [15], in India 62 % [24] and 82 % [25]. The variation might be due to the difference in study design, data collection tool, sample size and difference in study participants.

One of the factors significantly associated with depression was HIV and TB co-infection. The finding is similar with the study conducted in Jimma, Ethiopia [26] and South Africa [27]. Being diagnosed with HIV, which is a terminal life-long disease associated with high levels of stigma may also lead to high rates of mental disorders [27]. Hence, TB/HIV co-infected patients can be at higher risk of common mental disorders as a result of stigma and discrimination by the society [28].

Patients who had perceived TB stigma were about 11 times more likely to have depression than their counterparts. This is similar with a study conducted in Pakistan [29]. Previous study has proven that presence of perceived stigma is highly associated with depression. People with perceived stigma may have a low self image and be socially isolated which may predispose them depression [30].

Furthermore, the study indicated that poor social support was significantly associated with depression. The finding is similar with other studies in Nigeria [23]. Lack of (poor) social support and somatic illness may lead to increased psychological distress. On the other hand, good social support is vital for those with good health in prevention of depression [31].

### Prevalence and factors associated with anxiety among patients with Tuberculosis

The study showed that the prevalence of anxiety among TB patients was 41.5 %. The finding is similar with the study conducted in Pakistan 46.2 % [11] and in Greece 40.67 % [13] but lower than the study conducted in Romania 72.88 % [12]. The variation might be due to the difference in data collection tool which was STAI scale, might over estimate anxiety symptoms among TB patients in Romania.

With respect to gender, being female was significantly associated with anxiety. Different studies showed that anxiety disorders are more common in females than males. Biological factors might contribute for the differences.

Similar to depression, HIV and TB co-infection was significantly associated with anxiety. A similar finding was observed in a study conducted in Jimma, Ethiopia [26]. TB/HIV co-infected patients can be at higher risk of common mental disorders because of stigma and discrimination by the society [28].

Anxiety was also significantly higher among patients who had perceived TB stigma. A similar finding was seen in a study conducted in Pakistan [29]. Previous study has proven that presence of perceived stigma is highly associated with anxiety. People with perceived stigma may have a low self image and be socially isolated which may predispose them anxiety [30].

Anxiety was significantly higher among patients who were in intensive phase of TB treatment than those of patients in continuation phase of treatment. This is due to the fact that the symptoms of tuberculosis tend to be prominent in the intensive phase where patients get relieved as they progress to the continuation phase of TB treatment This may indicate Patients in the continuation phase of anti-TB treatment, the physical and functional status of the patients could improve significantly which in turn brings improved mental health status of individuals.

Regarding to social support, anxiety was significantly higher among patients who had poor social support than patients who had good social support. Lack of (poor) social support and somatic illness (TB) may lead to increased psychological distress (mental disorders); on the other hand, good social support is vital for those with good health in prevention of anxiety [31].

The study also revealed that use of substance (khat, cigarette & alcohol) was significantly associated with anxiety. This could be due to the fact that anxious patients are more prone to use substances to relief themselves from the stress or anxiety symptoms. Many studies established that 25-50 % of people with substance (khat, cigarette & alcohol) use related problem meet diagnostic criteria of anxiety disorder some time in their life time [32]. For instance, one study conducted on smoking in relation to anxiety and depression showed, anxiety had strong association with cigarette smoking [33, 34].

## Conclusion

The prevalence of depression, anxiety and co-morbid depression and anxiety (40.6 %) among TB patients were high. Both depression and anxiety had statistically significant association with co-morbid HIV infection, perceived TB stigma and poor social support. Being female sex, current substance (khat, alcohol and cigarette) use and being on intensive phase of TB treatment had statistically significant association with anxiety only. TB clinics should develop guidelines to screen and treat depression and anxiety among TB patients. Further research on risk factors of depression and anxiety should be conducted to strengthen and broaden the current findings.

## Limitation of the study

This study was cross-sectional study design, it did not allow establishing a temporal relationship between depression and anxiety and significant associated factors like substance (khat, cigarette and alcohol) use. Additionally, no detailed validation study was done for the perceived TB-stigma scale and Oslo 3-item social support scale. Also, substance use related factor was not assessed by standard tool.
